# A modified minimally invasive technique for pelvic organ prolapse: transvaginal extraperitoneal single-port lateral suspension

**DOI:** 10.3389/fmed.2026.1877022

**Published:** 2026-09-21

**Authors:** Chengling Zhang, Huisheng Ge, Qiang Zhang, Li He

**Affiliations:** Department of Gynecology, Chengdu Women’s and Children’s Central Hospital, School of Medicine, University of Electronic Science and Technology of China, Chengdu, China

**Keywords:** extraperitoneal approach, lateral suspension, pelvic organ prolapse, transvaginal mesh, vNOTES

## Abstract

**Objective:**

This retrospective comparative cohort study aimed to evaluate an improved transvaginal extraperitoneal single-port lateral suspension procedure for the treatment of anterior compartment prolapse and paravaginal defects while preserving the advantages of minimally invasive surgery and maintaining abdominal wall esthetics.

**Methods:**

A retrospective comparative cohort study was performed on 43 cases treated in our hospital. Twelve patients who underwent the improved lateral suspension procedure were included in the improved group, whereas 31 patients who underwent traditional lateral suspension served as the control group. Patients in each group underwent their respective surgical procedures.

**Results:**

No patient in either group experienced accidental vascular injury or hemorrhage. In the improved group, two procedures were unsuccessful due to inadvertent peritoneal puncture and entry into the abdominal cavity, and these peritoneal defects were repaired using transvaginal natural orifice transluminal endoscopic surgery (vNOTES). No other intraoperative or postoperative complications were observed. There were no statistically significant differences between the two groups regarding operative time, length of hospital stay, or estimated blood loss. Both groups showed significant postoperative improvements in POP-Q, PFDI-20, and PFIQ-7 scores.

**Conclusion:**

Based on these preliminary results, this modified procedure appears to be feasible, safe, and effective for gynecologists experienced in vNOTES and pelvic organ prolapse surgery. However, its efficacy and safety require further validation through larger prospective studies with longer follow-up periods.

## 1 Introduction

Pelvic organ prolapse (POP) is a significant health concern among women. With an aging population, the demand for surgical treatment of POP continues to increase (1, 2). Although more than 120 surgical procedures for prolapse have been described, the optimal surgical approach remains controversial. Therefore, increasing attention has been paid to developing pelvic floor surgical techniques that combine efficacy, safety, and minimal invasiveness (3).

Laparoscopic lateral abdominal wall suspension surgery, first reported by Dubuisson et al., is an emerging surgical technique for treating POP (4). Compared with laparoscopic sacrocolpopexy, this procedure has a shorter learning curve and is technically less demanding (5). Consequently, it has become an increasingly favored option among gynecologists performing primary pelvic floor surgery and has gained substantial popularity in recent years. However, its efficacy in managing severe anterior compartment prolapse, particularly in patients with paravaginal defects, remains limited and continues to be debated in the field of pelvic floor surgery (6).

Natural orifice transluminal endoscopic surgery (NOTES) represents a major innovation in minimally invasive surgery (7). In recent years, transvaginal natural orifice transluminal endoscopic surgery (vNOTES) has been increasingly adopted in various gynecological surgeries. Currently, most surgeons experienced in single-port laparoscopy can perform various gynecologic procedures using the vNOTES approach (8), including pelvic floor surgery (9), and numerous studies have demonstrated its safety and effectiveness.

In this study, we introduce a novel minimally invasive procedure for POP using the vNOTES approach. Similar to traditional lateral suspension, two arms of the mesh are placed in the extraperitoneal spaces and suspended on the lateral abdominal wall. However, unlike the conventional procedure, the entire operation is performed extraperitoneally, and the remaining two arms are fixed to the middle of the pelvic fascia tendon arch. The mesh is flattened in the vesicovaginal space to repair pelvic floor prolapse. The technique combines the advantages of lateral suspension and transvaginal mesh (TVM) implantation, providing a potential new surgical option for the treatment of POP.

## 2 Methods

### 2.1 General information

This retrospective study was conducted at Chengdu Women's and Children's Central Hospital from April 2024 to October 2025 and was approved by the Ethics Committee of Chengdu Women's and Children's Central Hospital (Approval No. 2026[76-2]). All hospitalized patients routinely provided written informed consent for the use of their anonymized clinical data for research and teaching purposes. All patients with pelvic organ prolapse were managed through the standardized system of the Chengdu Pelvic Floor Disease Center, and complete outpatient follow-up records were available for review. All patients signed the surgical consent form before surgery.

As the modified surgery involves transvaginal mesh implantation, its eligibility criteria were determined according to the indications for TVM surgery. To ensure patient safety, only patients aged 55 years or older were eligible for the modified procedure, and sexually active patients were excluded. The remaining indications and contraindications are consistent with those for traditional lateral suspension: (1) POP-Q stage Ⅱ–Ⅲ middle compartment prolapse, with or without associated vaginal wall prolapse; (2) exclusion criteria included serious medical comorbidities or complete closure of the rectovaginal pouch; and (3) a history of cesarean and pelvic surgery was not considered a contraindication. Twelve patients underwent the improved lateral suspension procedure and followed up. Thirty-one patients who received traditional lateral suspension were selected as the control group. Data collected included age, body mass index (BMI), history of pelvic surgery, pregnancy and delivery history, operative time, intraoperative blood loss, pain scores, and other indicators. The POP-Q scores (Ba, Bp, and C), PFDI-20 (Pelvic Floor Distress Inventory-20) scores, PFIQ-7 (Pelvic Floor Impact Questionnaire-7) scores were recorded preoperatively and at 3 months postoperatively for comparison. We decided that the surgery was successful if the patient met all of the following three criteria simultaneously; otherwise, it was classified as recurrence or failure: (1) the maximum distance of the anterior and posterior vaginal wall prolapse from the hymen was ≤0 cm, and the apical drop distance was ≤1/2 of the total vaginal length; (2) resolution of POP symptoms, as assessed by PFDI-20 Question No. 3 (“Do you usually see or feel something falling out in your vagina?”); and (3) no reoperation or pessary treatment for prolapse.

All operations were performed by a single surgeon to eliminate interoperator variability. The surgeon(LH) in our hospital has extensive experience in laparoscopic and POP surgery, having completed hundreds of vNOTES surgeries independently, including adnexectomy, hysterectomy, and lymphadenectomy for malignant tumors. Her surgical technique is stable and consistent.

### 2.2 Preoperative preparation

Because the procedure may involve posterior vaginal or perineal laceration repair, and bowel preparation is considered a precautionary measure, patients in both groups were given 90 mL of sodium phosphate 1 day before surgery. The vagina was rinsed twice with diluted iodophor solution. Intravenous administration of cefazolin sodium or cefmetazole sodium were performed 30 min before surgery and continued for 48 h postoperatively to prevent infection.

### 2.3 Surgical method

The improved lateral suspension procedure was performed via transvaginal extraperitoneal single-port laparoscopy using Titanized Mesh Implants TiLOOP Total 4 (T4 mesh). Patients underwent the following surgical procedures, as shown in Supplementary Videos S1–S3.

1. After disinfecting the cervix and vagina, the cervix was pulled outward. Following the injection of a water cushion, the anterior vaginal wall was incised longitudinally. The vesicovaginal space was entered, and the incision was extended upward to the urethrovesical junction and downward to the vesicocervical junction.
2. The lateral bladder space was dissected to reach the tendinous arch of the pelvic fascia.
3. The extraperitoneal space was entered via the lateral bladder space, and a laparoscopic port (PORT) was placed to establish extraperitoneal pneumoperitoneum.
4. The right extraperitoneal space was dissected toward the puncture point beneath the lateral abdominal wall. A small incision was made at a point located 4 cm above and 3 cm lateral to the bilateral anterior superior iliac spines.
5. Under extraperitoneal laparoscopic guidance, forceps were used to puncture into the extraperitoneal space and grasp mesh arm A. The same steps were repeated on the left side for mesh arm B (Figure 1A).
6. The vaginal portion of the mesh was flattened, and the lower portion was secured to the cervix using interrupted non-absorbable sutures.
7. Additional puncture points were marked at the intersections of a line 2 cm above the urethra and the bilateral inguinal folds.
8. The remaining two mesh arms (C and D) were suspended in the middle of the bilateral tendinous arches of the pelvic fascia using a puncture device.
9. The mesh was spread flat in the vesicovaginal space (Figures 1B,C), and the upper portion was secured to the vesicocervical fascia with interrupted non-absorbable sutures. The anterior vaginal wall incision was closed.
10. The four mesh arms were then simultaneously tensioned to maintain the mesh in a tension-free state and ensure that the cervix was positioned above −5.
11. Gauze rolls were packed after vaginal disinfection.

**Figure 1:**
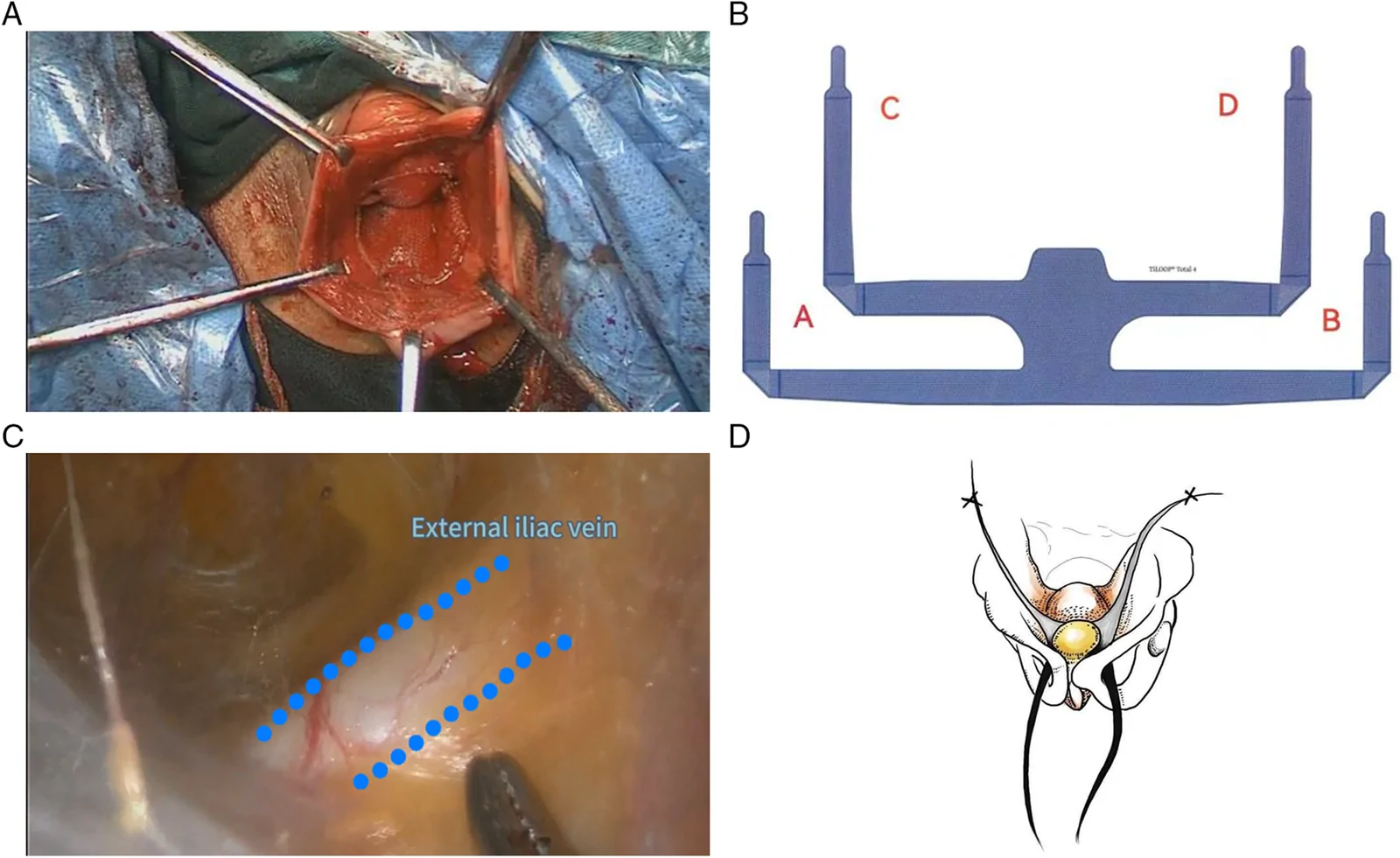
**(A)** Schematic diagram for four mash arms. **(B)** The mesh was spread flat in the vesicovaginal space. **(C)** A hand-drawn illustration showing the mesh and four arms. **(D)** The working space was located above the external iliac vessels.

The traditional lateral suspension procedure was performed via laparoscopy using the same mesh. Both procedures were performed with or without hysterectomy, vaginal wall repair, and perineal laceration repair, depending on the patient's prolapse.

### 2.4 Statistical analysis

IBM SPSS 22.0 software was used for statistical analysis. Normally distributed continuous data were expressed as the mean (standard deviation) and compared using an independent-samples or paired sample *t*-test (the effect size was calculated using Hedges *g* method). Non-normally distributed data were compared using the Mann–Whitney *U*-test or the Wilcoxon signed-rank test (*r* was used to calculate the effect size). Categorical data were described as counts (%), and comparisons were made using the chi-square test or Fisher's exact test, as appropriate [with effect sizes calculated using the odds ratio (OR) or Cramer's *V*]. *P* < 0.05 was considered statistically significant.

## 3 Results

The mean age of all patients was 53.3 years (range 34–70 years). There was a statistically significant difference in age between the two groups due to the differing inclusion criteria. No significant differences were observed between the groups regarding BMI, history of pelvic surgery, comorbidities, number of pregnancies, number of deliveries, or menopausal status (*P* > .05), as presented in Table 1.

**Table 1:** Preoperative demographic and clinical characteristics of the two groups.

Variable	Value (improved group)	Value (control group)	*t*/*u*/ *χ*^2^	*P*	Effect size	95% CI
Age (years), mean (SD)	59.92 (4.78)	50.81 (8.90)	3.346	0.002	1.12	0.41 to 1.81
BMI (kg/m^2^), mean (SD)	25.25 (2.64)	24.99 (2.37)	0.314	0.755	0.10	−0.55 to 0.76
Prior surgery, *n* (%)			0.658	0.814	0.113	0.0877 to 0.4019
No prior surgery	6 (50.0%)	17 (54.8%)				
Prior POP surgery	0 (0.0%)	1 (3.2%)				
Prior other surgery	6 (50.0%)	13 (41.9%)				
Number of pregnancies	3 (2–4)	3 (2–4)	181.500	0.901	0.019	0.0044 to 0.32
Number of deliveries	2 (1–2)	2 (1–2)	167.500	0.582	0.084	0.0088 to 0.35
Menopausal status, *n* (%)			3.671	0.055	0.1152	0.0024 to 0.9744
Premenopausal	1 (8.3%)	14 (45.2%)				
Postmenopausal	11 (91.7%)	17 (54.8%)				
POP-Q stage, *n* (%)			0.461	0.497	0.4696	0.0683 to 2.3947
Ⅱ	3 (25.0%)	13 (41.9%)				
Ⅲ	9 (75.0%)	18(58.1%)				

Patients in the two groups underwent different surgical procedures, and no patient suffered accidental vascular injury or hemorrhage. In the improved group, we defined technical success as completion of the procedure entirely via the transvaginal extraperitoneal single-port approach. Under this definition, 10 out of 12 patients (83.3%) achieved technical success. Two procedures were unsuccessful due to inadvertent peritoneal puncture into the abdominal cavity, and the operations were completed via vNOTES after repairing the ruptured peritoneum. Perioperative examination results were normal for all patients, and no patients developed postoperative fever (temperature ≥38 °C), hematoma, or poor wound healing. There were no significant differences in operative time, estimated intraoperative blood loss, concomitant procedures, or mean postoperative hospital stay, as presented in Table 2. The POP-Q (Ba, Bp, and C), PFDI-20, and PFIQ-7 scores were significantly improved postoperatively in both groups, as presented in Table 3. As a short-term observation, no mesh exposure or recurrence had been found so far.

**Table 2:** Postoperative data of the two groups.

Variable	Value (improved group)	Value (control group)	*t*/*u*/*χ*^2^	*P*	Effect size	95% CI
Postoperative follow-up (months), median (range)	6.5 (6.0–7.8)	11.0 (8.0–20.0)	54.000	<0.001	0.548	0.36 to 0.72
Operative time (min), mean (SD)	151.75 (61.78)	179.36 (47.41)	−1.572	0.124	−0.52	−1.19 to 0.14
Estimating blood loss intraoperative (mL), median (range)	50.0 (20.0–50.0)	50.0 (20.0–100.0)	148.000	0.289	0.159	0.0071 to 0.42
Length of hospital stay (days), median (range)	3.0 (3.0–4.0)	4.0 (3.0–5.0)	120.000	0.055	0.290	0.04 to 0.53
Postoperative pain score on the first day	1.0 (1.0–2.0)	2.0 (1.0–3.0)	125.500	0.081	0.264	0.02 to 0.52

**Table 3:** Comparison of pre- and postoperative outcomes for the two groups.

Improved group						
POP-Q point	Preoperative median (Q1, Q3)	Postoperative median (Q1, Q3)	*Z*	*P*	Effect size	95% CI
Ba	2.0 (−0.1, 2.0)	−3.0 (−3.0, −3.0)	−3.074	0.002	0.876	0.874 to 0.893
Bp	−0.3 (−2.0, 1.4)	−3.0 (−3.0, −3.0)	−2.810	0.005	0.797	0.665 to 0.88
C	2.0 (0.4, 2.0)	−5.0 (−5.0, −4.3)	−3.072	0.002	0.876	0.876 to 0.892
PFDI-20	43.72 (31.25, 69.23)	0.00 (0.00, 3.90)	−3.061	0.002	0.872	0.872 to 0.879
PFIQ-7	23.69 (14.46, 45.96)	0.00 (0.00, 3.57)	−2.943	0.003	0.838	0.674 to 0.877

Control group						
POP-Q point	Preoperative median (Q1, Q3)	Postoperative median (Q1, Q3)	*Z*	*P*	Effect size	95% CI
Ba	1.0 (0.0, 1.5)	−3.0 (−3.0, −3.0)	−4.728	<0.001	0.847	0.805 to 0.88
Bp	−2.0 (−3.0, −1.0)	−3.0 (−3.0, −3.0)	−3.459	0.001	0.619	0.383 to 0.771
C	2.0 (−1.0, 5.0)	−5.0 (−6.0, −5.0)	−4.872	<0.001	0.873	0.873 to 0.878
PFDI-20	42.85 (16.67, 71.00)	0.00 (0.00, 3.13)	−4.861	<0.001	0.871	0.871 to 0.873
PFIQ-7	32.00 (14.28, 57.13)	0.00 (0.00, 0.00)	−4.841	<0.001	0.868	0.85 to 0.872

Changes from pre- and postoperation between groups						
POP-Q point	Improved group median (Q1, Q3)	Control group median (Q1, Q3)	*Z*	*P*	Effect size	95% CI
Ba	−4.5 (−5.0, −2.8)	−3.5 (−4.5, −3.0)	−1.672	0.094	0.253	0.01 to 0.57
Bp	−2.8 (−4.38, −1.0)	−1.0 (−2.0, 0.0)	−1.872	0.061	0.283	0.03 to 0.55
C	−6.8 (−7.75, −6.0)	−7.5 (−10.0, −5.0)	−1.132	0.258	0.171	0.0098 to 0.42
PFDI20	−42.7 (−69.2, −25.8)	−42.9 (−71.0, −16.7)	−0.379	0.705	0.056	0.0058 to 0.34
PFIQ-7	−23.3 (−44.8, −14.5)	−32.0 (−57.1, −14.3)	−0.921	0.357	0.138	0.0083 to 0.38

## 4 Discussion

Laparoscopic lateral suspension surgery has been controversial since its introduction. Dubuisson et al. followed 417 patients who underwent lateral suspension for an average of 7.2 years. The results showed that anatomic success rates were 91.6% for the anterior compartment, 93.6% for the apical compartment, and 85.3% for the posterior compartment. The complication rate of Clavien–Dindo grade III or higher was 2.2% (10), supporting the efficacy and safety of the technique. Another controlled trial concluded that lateral abdominal wall suspension had a shorter learning curve than sacral fixation (5). Therefore, it is easy for gynecologists experienced in laparoscopic surgery to get started. Furthermore, Veit-Rubin et al.'s study showed that lateral abdominal wall suspension is safe and feasible both with and without hysterectomy (11). Transverse suspension through the lateral wall of the mesh follows the original anatomical structure of the uterus, so it may provide a more physiological vaginal axis direction than sacral fixation, which is also an important factor contributing to the high sexual satisfaction of patients after lateral suspension (12).

Our improvement technique was based on the traditional lateral suspension. The procedure was performed under continuous laparoscopic monitoring, with the working space located above the uterine vessels, the internal and external iliac vessels, and nerves (Figure 1D). Tactile feedback from the abdominal instruments helps localize the puncture point, and instrument movement is directly visualized via laparoscopy. The instruments penetrated the fat and muscle of the abdominal wall and entered the extraperitoneal space under direct visualization, ensuring operative safety. We compared the modified lateral suspension with the traditional transumbilical laparoscopic lateral abdominal wall suspension and found no statistically significant differences between the two groups in various postoperative outcomes. Although all 12 patients in the improved group underwent successful extraperitoneal mesh implantation, two procedures experienced inadvertent peritoneal puncture into the abdominal cavity, requiring repair of the ruptured pituitary via vNOTES. Therefore, we emphasize that not all doctors can perform this surgery. Only those with extensive experience in pelvic floor surgery and vNOTES surgery can successfully navigate from the lateral vaginal vault into the extraperitoneal space. Although we still completed the operations as planned and the patient experienced no discomfort during the postoperative follow-up, such technical breaches could potentially increase the risk of intraperitoneal mesh exposure in the future.

Nevertheless, we speculate that our modified procedure may offer several advantages over traditional procedures. The laparoscopic portion is completed entirely without entering the abdominal cavity, which theoretically may reduce surgical risk in elderly patients with compromised cardiopulmonary function. In particularly obese patients, the transvaginal extraperitoneal approach avoids interference from fat, intestines, and omentum, potentially shortening operative time. Only two small incisions are required in the lateral lower abdomen, which reduces the abdominal scar and results in superior cosmetic outcomes compared with traditional lateral suspension. Although the vaginal mucosa was opened during the procedure, allowing for more effective correction of anterior compartment prolapse and paravaginal defects than conventional lateral suspension. Because the mesh is fixed to the cervix, the effect of this surgery on middle pelvic prolapse may be superior to TVM. However, these advantages are conjectures, and more trial data are needed.

However, certain limitations remain. This procedure incorporates TVM implantation, which carries a certain risk of vaginal mesh exposure. A multicenter randomized controlled trial published in *JAMA* in 2024 reported a 36-month follow-up of 115 patients, with a vaginal mesh exposure rate of 5% (13). A systematic review indicated that the mesh erosion rate of lateral suspension varied across reports, ranging between 0% and 13%, likely due to differences in sample size, follow-up length, prosthetic material type, and the kind of concomitant surgical procedures (12). Therefore, although transvaginal mesh implantation remains a feasible option for selected patients with moderate to severe pelvic organ prolapse, careful patient selection and long-term surveillance are essential. In our modified procedure, although the vaginal mucosa is incised, the incision is located close to the cervix. We have further refined the technique by using a transverse incision 0.5 cm above the vesicocervical junction instead of a longitudinal incision in the anterior vaginal wall, which may help reduce the risk of mesh exposure. To date, no cases of mesh exposure have been observed in our cohort. Our preliminary results suggest that the modified surgery may be safe and feasible. However, the current median follow-up of the improved group was only 6.5 months, which is insufficient to establish long-term mesh safety. Therefore, it requires validation through larger prospective studies with longer follow-up periods. Because this modified surgery incorporates transvaginal mesh implantation, a minimum age threshold of 55 years was applied to ensure patient safety, and sexually active patients were also excluded. The age difference among patients may lead to selection bias and may impact the results.

## 5 Conclusions

Based on these preliminary results, this modified procedure appears to be feasible, safe, and effective for gynecologists experienced in vNOTES and pelvic organ prolapse surgery. However, its efficacy and safety require further validation through larger prospective studies with longer follow-up periods.

## Data Availability

The raw data supporting the conclusions of this article will be made available by the authors, without undue reservation.
